# The Role of the Melatoninergic System in Light-Entrained Behavior of Mice

**DOI:** 10.3390/ijms18030530

**Published:** 2017-03-01

**Authors:** Martina Pfeffer, Horst-Werner Korf, Helmut Wicht

**Affiliations:** 1 Dr. Senckenbergische Anatomie II, Fachbereich Medizin, Goethe-Universität Frankfurt, Theodor-Stern-Kai 7, D-60590 Frankfurt am Main, Germany; Korf@em.uni-frankfurt.de (H.-W.K.); wicht@em.uni-frankfurt.de (H.W.); 2 Dr. Senckenbergisches Chronomedizinisches Institut, Goethe-Universität Frankfurt, Theodor-Stern-Kai 7, D-60590 Frankfurt am Main, Germany

**Keywords:** circadian, stability, chronotype, melatonin, locomotor rhythm, diurnal

## Abstract

The role of endogenous melatonin for the control of the circadian system under entrained conditions and for the determination of the chronotype is still poorly understood. Mice with deletions in the melatoninergic system (melatonin deficiency or the lack of melatonin receptors, respectively) do not display any obvious defects in either their spontaneous (circadian) or entrained (diurnal) rhythmic behavior. However, there are effects that can be detected by analyzing the periodicity of the locomotor behaviors in some detail. We found that melatonin-deficient mice (C57Bl), as well as melatonin-proficient C3H mice that lack the melatonin receptors (MT) 1 and 2 (C3H MT1,2 KO), reproduce their diurnal locomotor rhythms with significantly less accuracy than mice with an intact melatoninergic system. However, their respective chronotypes remained unaltered. These results show that one function of the endogenous melatoninergic system might be to stabilize internal rhythms under conditions of a steady entrainment, while it has no effects on the chronotype.

## 1. Introduction

The circadian rhythmogenetic system regulates many behavioral and physiological processes and its disruption can thus lead to many different health problems. Continued disturbances of the rhythmogenetic system are linked to depression, insomnia, cardiovascular disease, cancer and even premature death [1,2,3,4,5]. Aging, shift work, jet lag, light pollution or social jet lag have destabilizing effects upon the rhythmogenetic system [1,6,7,8,9,10]. Generally, such disturbances lead to fragmented, unstable locomotor/activity patterns in mice and men [6,9,10,11,12,13].

Melatonin is synthesized at night by the pineal organ. It encodes the phase and the length of the dark period and thus transduces photoperiodic information [14]. Melatonin affects the endogenous clock; the hormone and its agonists can therefore be used therapeutically to treat desynchronization of circadian rhythms. The hormone appears as a promising treatment for sleep- and circadian-rhythm related disorders [15]. Indeed, melatonin aids mice and men in a more rapid re-entrainment to a new light regimen after exposure to jet lag [16,17], but the role of melatonin under conditions of a more regular, physiological entrainment, is unknown.

Our studies are focused on the role of melatonin under conditions of a steady entrainment. It is known that melatonin is neither sufficient nor necessary for the generation of diurnal or circadian rhythmicity in mice. Melatonin-deficient mice, such as the C57Bl strain [18,19] display rhythmic behaviors, be it under free-running or entrained conditions. Kasahara et al. [20] thus concluded that there are “no apparent effects of melatonin on circadian behaviors”—but, as we will show, such effects exist.

We have recently developed methods that permit us to analyze changes/differences in the dynamics of the diurnal behavior of mice, in particular we can now determine chronotypes and estimate the stability/lability of the behavioral locomotor rhythms over several days [9,10,21]. We used these methods to investigate the role of melatonin under conditions of a steady entrainment. We investigated the locomotor activity rhythms of melatonin-deficient mice (C57Bl), melatonin-proficient mice (C3H), and C3H mice with targeted deletion of the melatonin receptor 1, MT1, (C3H MT1 KO), the melatonin receptor 2 ,MT2, (C3H MT2 KO), or of both receptor types (C3H MT1/2 KO) under 12 h light/12 h darkness (LD).

## 2. Results

**Figure 1:**
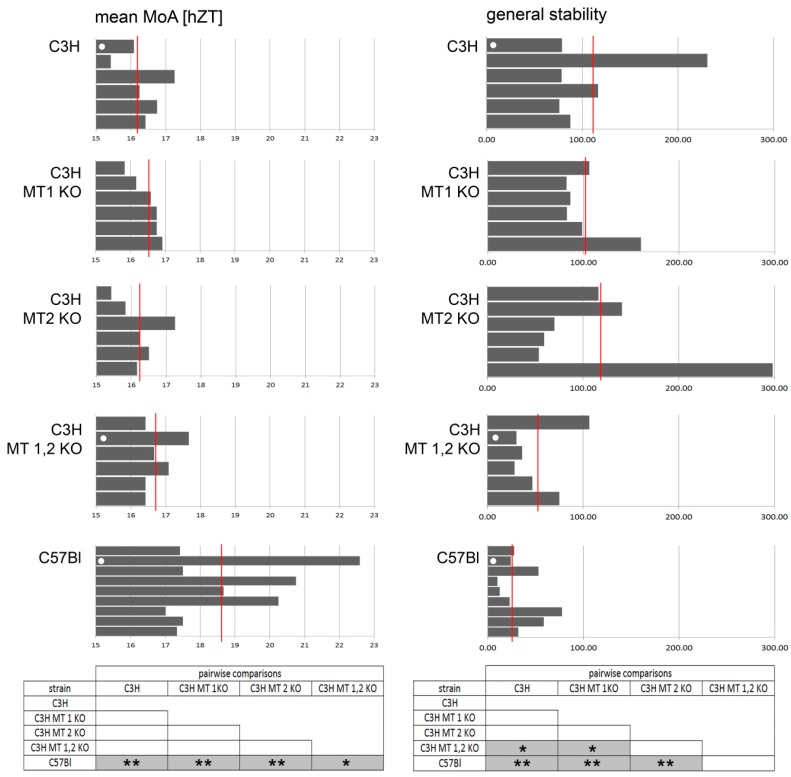
Chronotype (mean MoA) and general stability in interstrain comparisons. Each bar in the histograms represents a single mouse; the red lines indicate the mean values. The white dots in the bars indicate individual mice whose actograms are shown in Figure 2. The pairwise interstrain comparisons shown in boxes below are Bonferroni–Holm corrected Conover–Iman comparisons that were carried out on the sets of data stemming from the individual strains; shaded boxes with asterisks (* *p* < 0.05; ** *p* < 0.01)indicate significant differences between the respective strains. Prior to these pairwise tests, Kruskal–Wallace tests were applied and they confirmed (*p* < 0.01) the inhomogeneity of the strains with respect to the measurement parameters.

**Figure 2:**
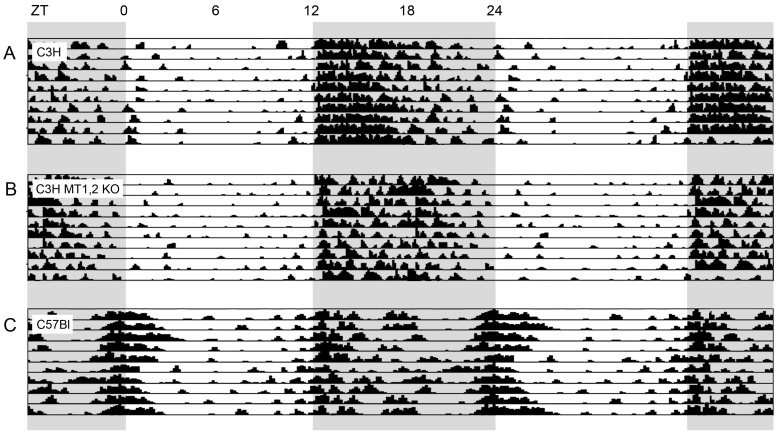
Double plotted actograms of a C3H mouse (**A**); and a MT 1,2 double KO mouse (**B**); and a C57Bl mouse (**C**). Grey indicates darkness.

The C3H, the C3H MT KOs and the C57Bl did not differ with regard to their total locomotor activity. However, there were significant differences in the chronotype and the general stability. Figure 1 presents the numerical and statistical summary of the data. C57Bl mice have a significantly later chronotype than the C3H, (see left box “pairwise comparisons” in Figure 1) mainly due to the fact that C57Bl display a second activity peak around zeitgeber time (ZT) 24 (Figure 2). They also had, in comparison to C3H, a relatively unstable locomotor rhythm (Figure 1).

Among the C3H-mouse strains, deletion of one or both melatonin receptors did not influence the chronotype, but the deletion of both MT receptors affected the general stability (Figure 1). The C3H MT 1,2 double knockout mice had significantly less stable behavioral rhythms than the other C3H-derived strains and were, in this respect, statistically indistinguishable from the C57Bl mice (see right box “pairwise comparisons” in Figure 1).

Figure 2 shows raw actograms of individual mice (identified by the white dots in the corresponding bars of Figure 1). The upper actogram (A) is from a C3H mouse. It displays the “early peak/slow decline” pattern that is typical of the entire strain. The C3H MT1,2 KO mouse shown in (B) retains this pattern, but is obviously much less stable regarding its daily repetition. The C57Bl-mouse in (C) shows the typical “twin peak” pattern of locomotor activity with a second activity bout that extends well into the light phase.

## 3. Discussion

Our studies are focused on the (behavioral) role of the endogenous melatoninergic system under conditions of a regular, steady, quasi-nycthemeral entrainment. As shown here and in previous studies [17,20,22], an intact melatoninergic system is not required for the generation of diurnal rhythmicity in mice. The same holds true in humans. In (rare) cases of an absence of rhythmic melatonin secretion from the pineal gland, the individuals nevertheless do entrain to a diurnal sleep/rest pattern [23].

However, as shown here, melatonin deficiency or the deletion of both its receptors decrease the general stability of locomotor rhythms under entrained conditions in mice.

The effect is mediated by MT 1 and MT 2 receptors; both need to be knocked out in order to destabilize the rhythms in C3H mice. An inspection of the numerical values of the “general stability” shows that the effect, in fact, seems to be almost entirely mediated by these two receptors. Knocking out both of them brings the C3H MT 1,2 KO mice “down” to the low level of stability of C57Bl mice and the two strains are, in that respect, statistically indistinguishable.

Given the wide distribution of MT-receptors throughout the body and the brain [24,25], the synchronizing action of melatonin might influence central as well as peripheral oscillators. It is, in fact, unlikely that the synchronizing effect of melatonin acts exclusively at the level of the master clock in the suprachiasmatic nucleus. The latter expresses only MT1 receptors [25], and, as we have shown, the knockout of that receptor alone does not affect the robustness of the behavioral rhythms.

One may ask—not from a pathological, but from a biological and evolutionary point of view—what a robust rhythm is good for or why an unstable one should be disadvantageous. It is possible, but not proven, that beings with stable rhythms are less susceptible to circadian disruptions. On the other hand, unstable rhythms may, in fact, be more malleable in response to environmental challenges and thus be advantageous. C57Bl mice have unstable rhythms. Yet, as far as longevity and reproductive success are concerned, they are the most “successful” laboratory mice, they in fact live—on average—longer than the stable C3H [26].

In that vein, Daan et al. [27] have recently published a remarkable study of “lab mice in the field” under seminatural conditions. They showed that the reproductive success of mice with genetic lesions in the molecular clockwork is by no means lower than that of intact mice. Already in 1976, Pittendrigh and Daan [28] showed that the accuracy of the endogenous circadian pacemaker differs a lot between different species—hamsters have accurate clocks, mice generally have more unstable ones. Refinetti [29] found that gerbils have an even more accurate endogenous pacemaker than hamsters—yet they all (gerbils, hamsters and mice) live and reproduce with some success out in the field. Evidently, we need to learn much more about the significance of the effects that we observe in the laboratory for “real life”.

Notably, we did not observe any effect of the melatoninergic system on the chronotype, i.e., the general (and strain-specific) phase angle of entrainment. The MT1,2 KO mice maintained the same (early) chronotype and the same general activity pattern as their parent strain. Thus, the melatoninergic system does not determine the chronotype.

In summary, we have shown an effect of the melatoninergic system on the stability of diurnal rhythmic behavior in mice—melatonin enhances the timely accuracy of the daily reproduction of the strain-specific activity patterns and therefore improves timekeeping efficiency.

## 4. Material and Methods

### 4.1. Animals

The experiments reported here were conducted according to the policy on the use of Animals in Neuroscience Research and the Policy on Ethics and are approved by the Society for Neuroscience and by the European Directive 2010/63/EU. We used 33 adult (8–12 weeks old) male mice from the following strains:

(1) C57Black/6J (short: C57Bl) mice. These mice (*n* = 9) were obtained from Charles River Europe (Sulzfeld, Germany). They are melatonin-deficient [18] and display a late chronotype and a relatively unstable behavioral rhythm [21].
(2) C3H/HeN-Pde6b^rd1−,−^ (short: C3H). These mice (*n* = 6) are melatonin-proficient [18] and display an early chronotype and a very stable behavioral rhythm [21].
(3) C3H/HeN-Pde6b^rd1−,−/mt1−,−/mt2−,−/mt1,2−,−^ (short: C3H MT 1 KO, *n* = 6/C3H MT 2 KO, *n* = 6/C3H MT 1,2 KO, *n* = 6). Mice with a targeted deletion of the MT1 gene (C3H MT1 KO, [30]) and the MT2 gene (C3H MT2 KO, [31]) were bred on a melatonin-proficient C3H/HeN-Pde6b^rd1−,−^ background for at least 10 generations. These single KO mice were kindly provided by Dr. David Weaver (UMASS Medical School, Worcester, MA, USA). C3H MT1,2 KO double deficient mice were obtained in our lab by crossing C3H MT1 KO and C3H MT2 KO mice and breeding the MT1/2 double KO offspring for at least 10 generations [17].

### 4.2. Animal Housing, Entrainment and Data Recording

#### 4.2.1. Housing and Entrainment

The animals were kept in individual cages. They were adapted to a 12 h light (L):12 h dark (D) cycle (light phase: 230 µW/cm^2^; dark phase: dim red light; <5 µW/cm^2^) for at least 12 days before the experiments. They had access to food and water ad libitum. The 12:12 LD light regimen was maintained throughout all experiments reported here. Lights-off was defined as [hZT] 12.

#### 4.2.2. Locomotor Activity/Actograms

The general spontaneous locomotor activity was monitored using infrared motion detectors (Mouse-E-Motion, Hamburg, Germany); activity was continuously recorded during the experiments in 10-min intervals. The Clocklab software (Actimetrics, Wilmette, IL, USA) was used for data handling and generation of double-plotted actograms.

### 4.3. Measurement Parameters Derived from the Actograms

The MoA (“median of activity”) is a numerical (in [hZT]) measurement of the chronotype in the presence of an external zeitgeber. The daily MoA is that point in time at which a mouse has accomplished 50% of its daily “locomotor work” after the onset of the main activity phase. For the mouse strains used in this study, the onset of the main activity phase is at [hZT] 12, i.e., at “lights off”. The mean of the daily MoAs over an observation period of several days (mean MoA) permits to differentiate “late” and “early” mice and can thus be used as a measure for the chronotype. Since the MoA can be determined for each single day, its standard deviation over the observation period (SDevMoA) is a measure of the accuracy of the daily reproduction of the chronotype: a low SDevMoA will indicate stable chronotypes. We used a self-written software (programmed in Excel) to determine the daily and mean MoAs and their standard deviations from the raw data [10,21].

The Qp-values [29] are another measure for the robustness of behavioral rhythms. In short, the Qp-values quantify the deviation of the behavioral rhythm from an ideal, stationary time series with unchanging period length. High Qp-values (scaled in 0%–100%) will indicate stable rhythms. We used the Website of Refinetti’s lab (see below, ref. [29]) to calculate these values.

As shown previously [21], there is a strong inverse correlation between the Qp-values and the SDevMoA. Thus, the two values may be combined (Qp/SDevMoA) to a measure of “general stability” (in [arbitrary units]). In addition, we measured the total activity, but generally found no significant differences between the mice/strains investigated here.

### 4.4. Experimental Design and Statistics

We compared (“interstrain comparisons”; 33 animals, see Table 1) the behaviors of mice of different strains. The daily and mean MoAs and the values for the general stability (Qp/SDevMoA) were determined for each mouse and the data were pooled for each strain. Kruskal–Wallace tests for homogeneity with subsequent pairwise Conover–Iman comparisons (Bonferroni–Holm corrected) were applied to probe the likelihood of the differences observed between the strains.

All statistical analyses were carried out using the BIAS-program package [32]; significance levels were set to <0.05 (single asterisk in Figures) and <0.01 (double asterisks).

## Figures and Tables

**Table 1:** Raw data and statistical methods. Bullets: Actograms from these mice are shown in Figure 2.

Measurement over 10–12 Days. Interstrain Comparisons of mean MoA and General Stability via Kruskal–Wallace/Conover–Iman Tests	Mean MoA [hZT]	SDev MoA [hZT]	Qp [%]	General Stability [Arbitrary Units]
**C3H**				
(mouse ID)				
(19) ●	16.10	0.4691	37.0	78.8
(30)	15.41	0.1969	45.4	230.6
(4)	17.25	0.4369	34.2	78.3
(3)	16.25	0.3379	39.4	116.6
(2)	16.75	0.4983	37.9	76.9
(1)	16.42	0.3935	34.4	87.4
**C3H MT 1 KO**				
(mouse ID)				
(17)	15.83	0.3878	41.1	106.0
(16)	16.17	0.4522	37.2	82.3
(15)	16.58	0.4931	42.5	86.2
(13)	16.75	0.4397	36.3	82.6
(24)	16.75	0.3809	37.5	98.4
(14)	16.92	0.2500	40.0	160.0
**C3H MT 2 KO**				
(mouse ID)				
(7)	15.42	0.3472	40.3	116.1
(6	15.83	0.3206	45.1	140.7
(18)	17.25	0.4346	30.4	69.9
(5)	16.25	0.6314	37.4	59.3
(8)	16.50	0.5503	29.7	53.9
(23)	16.12	0.1476	44.0	298.1
**C3H MT 1,2 KO**				
(mouse ID)				
(12)	16.42	0.3685	39.2	106.4
(20) ●	17.67	0.8963	27.3	30.5
(9	16.67	0.6432	23.4	36.4
(10)	17.08	0.8579	24.3	28.3
(11)	16.42	0.7010	32.9	46.9
(21)	16.42	0.3839	28.9	75.3
**C57Bl**				
(mouse ID)				
(16)	17.41	1.1429	31.5	27.6
(13) ●	22.58	1.1279	27.0	23.9
(11)	17.50	0.5294	28.1	53.1
(7)	20.75	1.8064	18.2	10.1
(12)	18.67	1.6379	20.5	12.5
(10)	20.25	1.0628	24.3	22.9
(26)	17.00	0.4625	35.8	77.4
(25)	17.50	0.5966	35.0	58.7
(27)	17.33	1.1021	35.4	32.1
